# Editorial: Synthetic Nucleic Acids for Expanding Genetic Codes and Probing Living Cells

**DOI:** 10.3389/fbioe.2021.720534

**Published:** 2021-07-02

**Authors:** Patrick O'Donoghue, Ilka U. Heinemann, Chenguang Fan

**Affiliations:** ^1^Department of Biochemistry, The University of Western Ontario, London, ON, Canada; ^2^Department of Chemistry, The University of Western Ontario, London, ON, Canada; ^3^Department of Chemistry and Biochemistry, University of Arkansas, Fayetteville, AR, United States

**Keywords:** RNA, synthetic biology, genetic code expansion, fluorescent proteins, RNAi, microRNA, tRNA, non-canonical amino acids

Synthetic biology is a new and emerging discipline that involves the design and construction of novel biological probes and entities such as enzymes, genetic circuits, or synthetic cells with engineered or expanded biological functions. The field of synthetic biology developed as a result of revolutionary biotechnologies, such as high-throughput DNA synthesis, rapid genome sequencing, as well as directed and continuous evolution approaches to probe cellular functions at the most basic level, including modifying the sequences of nucleic acids and proteins in living cells. Re-writing or re-programming existing biological systems to produce designer proteins and nucleic acid polymers is a major area of focus in synthetic biology.

This special issue of *Frontiers in Bioengineering and Biotechnology* brings together diverse and cutting-edge applications in synthetic biology. The work highlights the utility, adaptability, and innovative potential of RNAs, including engineered or synthetic RNAs, in revealing biology at the molecular level. Applications of these synthetic nucleic acid technologies span multiple fundamental areas of synthetic biology including genetic code expansion and new fluorescent probes for observations of mistakes in protein synthesis or microRNA activity in live cells.

## Genetic Code Expansion With and Without Cells

Several of the studies in this special issue focused on the area of genetic code expansion. The field involves a range of methods that enable protein synthesis with additional or non-canonical amino acids (ncAA) beyond the standard 20 amino acids building blocks normally used in protein synthesis. Overall, research in this field has led to a staggering array of different chemical functionalities that have applications in site-specific protein labeling with fluorescent or reactive probes, and in protein modifications, such as programmed phosphorylation or acetylation. Indeed, as an introduction to this area, Chung et al. provided an insightful review of the many applications of genetic code expansion, highlighting studies that biochemically characterize specifically labeled or modified proteins.

Pyrrolysyl-tRNA synthetase (PylRS) is a naturally occurring enzyme in certain anaerobic archaea and bacteria that has been used to incorporate > 50 different ncAAs into proteins, reviewed in Wan et al. (2014). Jiang et al. pushed the enzyme yet further and produced an engineered PylRS mutant with an enhanced activity and the ability to incorporate additional ncAAs, including histidine, and cysteine analogs that have applications in protein engineering and studies of protein function.

Going beyond the barriers to synthetic biology associated with living cells, Cui et al. reviewed advanced cell-free methods for protein synthesis with multiple different ncAAs. Cell-free approaches enable the production of designer proteins with an expanded palette of ncAAs that include diverse chemical functionalities or unusual backbones (β-amino acids or α-hydroxy acids), including those that might be toxic to cells. Using a cell-free system, Xiao et al. developed a novel approach to generate synthetic proteins with site-specific modifications using a flexizyme. The method employs a flexizyme, which is a nucleic acid enzyme or ribozyme that aminoacylates transfer RNAs (tRNAs) with a desired non-canonical amino acid (ncAA). The authors demonstrated synthesis of histones with site-specific lysine acetylation or incorporation of a non-hydrolysable thioacetyl-lysine analog for studies on the histone code.

**Figure 1:**
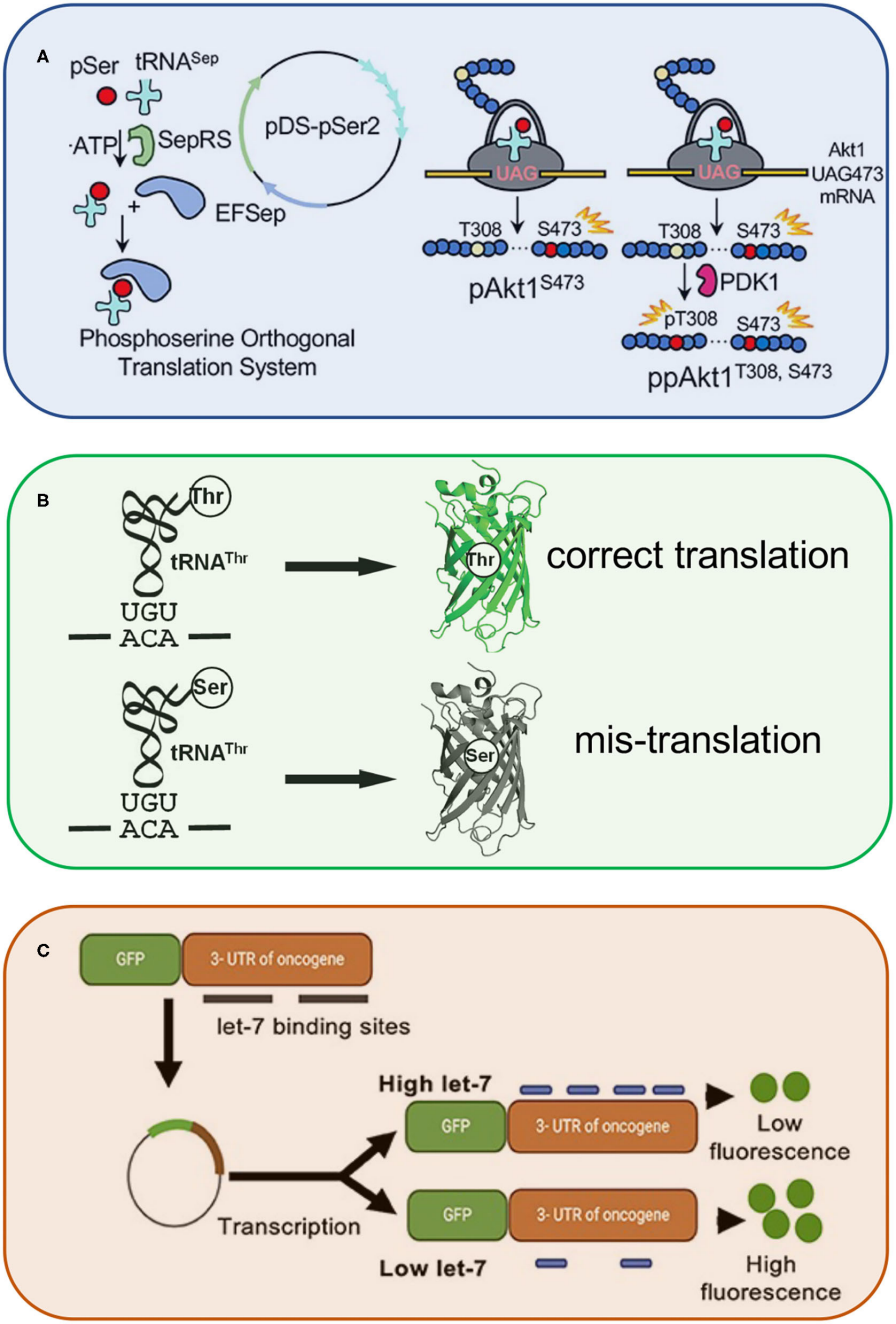
Synthetic and engineered RNAs for genetic code expansion and probing living cells. The schematic highlights a few of the innovative technologies described in this special issue. These include engineered transfer RNAs (tRNA^Sep^) to enable genetic code expansion and applications such as the production of active human kinases **(A)**. In addition, this special issue highlights novel reporters of RNA activity, including green fluorescent protein reporters sensitive to either tRNA-dependent amino acid mis-incorporation **(B)** or microRNA activity **(C)**.

Finally, McKenna et al. used genetic code expansion to generate specifically phosphorylated forms of the oncogenic kinase, AKT1 (Figure 1A). The system relies on an engineered transfer RNA (tRNA) (Hohn et al., 2006) that is specifically recognized by phosphoseryl-tRNA synthetase (SepRS) to reassign UAG codons to phosphoserine. The authors produced active variants of the kinase to validate putative AKT1 substrates (Balasuriya et al., 2020), which represent new targets to inhibit AKT1-dependent oncogenesis.

## Fluorescent Live-Cell Reporters for Mistranslation and Gene Silencing

Chen et al. engineered a novel fluorescent reporter (Figure 1B) to observe and quantify errors in proteins synthesis in live cells. The technology enabled studies of an editing defective tRNA synthetase that produces both properly aminoacylated as well as mis-aminoacylated tRNA species leading to mistranslation in bacterial cells.

MicroRNAs are naturally occurring small non-coding RNAs that bind to particular messenger RNAs (mRNAs) for gene silencing. Although the ability of miRNAs to regulate gene expression, including that of oncogenes, is well-known, approaches to measure microRNA activity are often lacking. Siddika and Heinemann reviewed a new and emerging field devoted to engineering protein reporters and new methods to detect and quantify microRNA activity, localization, and quantity (Figure 1C). Next generation RNA sequencing, for example, can uncover unknown microRNAs, and fluorescent microRNA probes can measure the level of *active* rather than total microRNA in living cells (Turk et al., 2018).

The ability to precisely manipulate gene expression is important for defining how gene products function and for designing genetic circuits for synthetic biology. Indeed, microRNAs inspired the use of synthetic RNAs to silence gene expression as well. RNA interference (RNAi) is a technology that allows targeted silencing of particular mRNAs with complementary sequences. Because silencing RNAs (siRNAs) are normally produced as a duplex, the opposite or passenger strand, has significant potential to silence off-target genes. Sheng et al. reviewed new approaches to reduce off-target gene silencing using single stranded siRNAs.

In summary, here we highlighted innovate approaches involving engineered or synthetic nucleic acid-based technologies to expand the genetic code with additional amino acids or to observe or manipulate the activity of different kinds of RNAs in living cells. We anticipate that the studies contained in our special issue will inspire new RNA technologies for applications in biomedical research and synthetic biology.

## Author Contributions

All authors listed have made a substantial, direct and intellectual contribution to the work, and approved it for publication.

## Conflict of Interest

The authors declare that the research was conducted in the absence of any commercial or financial relationships that could be construed as a potential conflict of interest.

## References

[B1] Balasuriya N. Davey N. E. Johnson J. L. Liu H. Biggar K. K. Cantley L. C.. (2020). Phosphorylation-dependent substrate selectivity of protein kinase B (AKT1). J. Biol. Chem. 295, 8120–8134. 10.1074/jbc.RA119.012425 32350110 PMC7294097

[B2] Hohn M. J. Park H. S. O'Donoghue P. Schnitzbauer M. Soll D. (2006). Emergence of the universal genetic code imprinted in an RNA record. Proc. Natl. Acad. Sci. U.S.A. 103, 18095–18100. 10.1073/pnas.0608762103 17110438 PMC1838712

[B3] Turk M. A. Chung C. Z. Manni E. Zukowski S. A. Engineer A. Badakhshi Y.. (2018). MiRAR-miRNA activity reporter for living cells. Genes 9:305. 10.3390/genes9060305 29921790 PMC6027049

[B4] Wan W. Tharp J. M. Liu W. R. (2014). Pyrrolysyl-tRNA synthetase: an ordinary enzyme but an outstanding genetic code expansion tool. Biochim. Biophys. Acta 1844, 1059–1070. 10.1016/j.bbapap.2014.03.002 24631543 PMC4016821

